# Post mortem findings and their relation to AA amyloidosis in free-ranging Herring gulls (*Larus argentatus*)

**DOI:** 10.1371/journal.pone.0193265

**Published:** 2018-03-01

**Authors:** Désirée S. Jansson, Caroline Bröjer, Aleksija Neimanis, Torsten Mörner, Charles L. Murphy, Faruk Otman, Per Westermark

**Affiliations:** 1 Department of Animal Health and Antimicrobial Strategies, National Veterinary Institute (SVA), Uppsala, Sweden; 2 Department of Pathology and Wildlife Diseases, National Veterinary Institute (SVA), Uppsala, Sweden; 3 Department of Disease Control and Epidemiology, National Veterinary Institute (SVA), Uppsala, Sweden; 4 Department of Medicine, University of Tennessee Medical Center, Knoxville, TN, United States of America; 5 Department of Immunology, Genetics and Pathology, Uppsala University, Uppsala, Sweden; Justus-Liebeig University Giessen, GERMANY

## Abstract

Since the late 1990s, high mortality and declining populations have been reported among sea birds including Herring gulls (*Larus argentatus*) from the Baltic Sea area in Northern Europe. Repeated BoNT type C/D botulism outbreaks have occurred, but it remains unclear whether this is the sole and primary cause of mortality. Thiamine deficiency has also been suggested as a causal or contributing factor. With this study, we aimed to investigate gross and microscopic pathology in Herring gulls from affected breeding sites in Sweden in search of contributing diseases. Herring gulls from Iceland served as controls. Necropsies and histopathology were performed on 75 birds, of which 12 showed signs of disease at the time of necropsy. Parasites of various classes and tissues were commonly observed independent of host age, e.g. oesophageal capillariosis and nematode infection in the proventriculus and gizzard with severe inflammation, air sac larid pentastomes and bursal trematodiasis in pre-fledglings. Gross and microscopic findings are described. Notably, amyloidosis was diagnosed in 93 and 33% of the adult birds from Sweden and Iceland, respectively (*p*<0.001), with more pronounced deposits in Swedish birds (*p*<0.001). Gastrointestinal deposits were observed in the walls of arteries or arterioles, and occasionally in villi near the mucosal surface. Amyloid was identified within the intestinal lumen in one severely affected gull suggesting the possibility of oral seeding and the existence of a primed state as previously described in some mammals and chickens. This could speculatively explain the high occurrence and previously reported rapid onset of amyloidosis upon inflammation or captivity in Herring gulls. Amyloid-induced malabsorbtion is also a possibility. The Herring gull SAA/AA protein sequence was shown to be highly conserved but differed at the N-terminus from other avian species.

## Introduction

Declining free-living wild avian populations have been reported from the Baltic Sea region since the 1990s, and the Herring gull (*Larus argentatus*) is one of the affected species [1]. The Baltic Sea is considered to be one of the most polluted seas in the world [2]. However, the impact of different threats such as anthropogenic pollutants, predation, oil pollution, changes in feed resources and diseases is not well understood [3]. Between 2000 and 2004 an estimated number of 10,000 Herring gulls died in the Blekinge Archipelago in south-eastern Sweden in a series of mortality events [4]. Dead and paralyzed Herring gulls from these outbreaks were repeatedly diagnosed with botulism type C or C/D [4,5]. Prior to these outbreaks, botulism was very rarely diagnosed in the wild avifauna of Sweden, and the epizootology and population impact of botulism remain poorly understood. Moreover, avian pasteurellosis outbreaks [3,6] and thiamine deficiency [7] have been reported from avian populations of the Baltic Sea.

Unexpectedly, amorphous eosinophilic materials consistent with amyloid deposits were observed microscopically in one-third of affected and unaffected Herring gulls investigated during an outbreak of botulism [4]. Systemic amyloidosis has long been recognized in captive animals, including birds [8,9,10,11,12]. Marine and coastal birds in captivity, especially those of the orders *Anseriformes*, *Gruiformes* and *Charadriiformes* (to which genus *Larus* belongs) seem to be particularly prone to develop amyloidosis. Captivity seems to be an obvious risk in birds and crowding of animals may be one contributing factor. It has been noted that the relative frequency of amyloidosis in captive *Anseriformes* was higher in a zoological collection of birds when the number of birds increased without corresponding increase of space [13]. In addition, articular amyloidosis occurs in gallinaceous birds [14].

Systemic amyloidoses is a heterogeneous group of disorders where a protein has misfolded and aggregated into fibrils with characteristic properties. In humans, 17 different proteins are presently known to aggregate in this way, each associated with a specific disease [15]. In birds, only AA amyloidosis is known to occur. AA amyloidosis is usually a consequence of longstanding inflammation. The main amyloid fibril component is protein AA, which is derived from the larger precursor serum AA (SAA) by removal of a C-terminal segment of varying length. SAA is a protein family with members expressed by several genes. In humans and mice, amyloid protein SAA1 and SAA2 are acute phase reactants mainly expressed by the liver. The human *SAA3* is a pseudogene and SAA4 is a constitutively produced protein expressed by several different tissues. Plasma concentration of acute phase SAA is persistently high in some chronic inflammatory diseases and this is a prerequisite for development of AA amyloidosis.

Like in mammalian AA amyloidosis, the avian form is systemic with deposits in many different organs except brain [12]. The clinical signs in humans are usually renal with proteinuria due to glomerular deposits, although other consequences may also develop. Clinical signs in systemic amyloidosis of birds seem to be few [10], but are probably difficult to evaluate.

Although there are some sporadic reports of amyloidosis in free-living wild birds [4,10,16,17], in-depth characterization of this process has not been done. Therefore, the present study was undertaken to investigate the occurrence and organ and tissue distribution of amyloid among Herring gulls from along the Swedish coast and in inland habitats. Gross and microscopic findings were compared to Herring gulls from an apparently healthy population in Iceland to identify any potential underlying factors for amyloidosis development. Additionally, the amino acid sequence of the Herring gull AA protein was determined.

## Material and methods

### Sampling and clinical assessment

**Fig 1:**
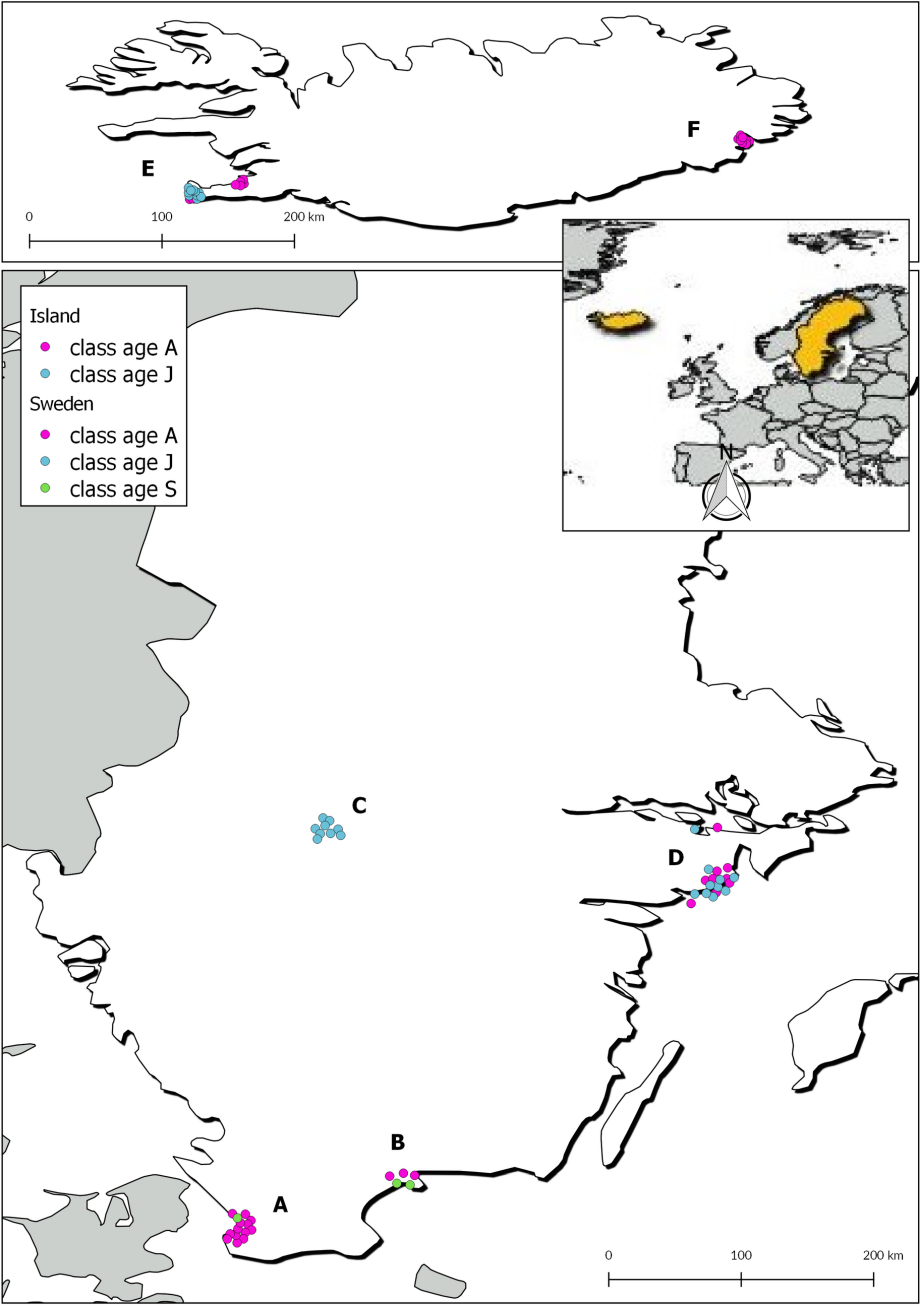
Maps of Sweden and Iceland showing collection sites of Herring gulls (*Larus argentatus*) included in the study. Adult birds are shown in pink, subadults in green and juveniles in blue. A = Malmö, Skåne, B = Blekinge Archipelago, C = Lake Vänern, D = Södermanland, E = South-western Iceland, F = Eastern Iceland. (Software QGIS, map ESRI Europe, projection EPSG:4326, WGS84). Reprinted from Data and Maps, 1998 under a CC BY license, with permission from ESRI Sverige, original copyright 1996, 1998.

In total, 75 Herring gulls (Table 1) were included in the study. Forty-six birds were collected during the summer of 2004 (12 May to 17 July) from 14 breeding sites in four areas in Sweden; Skåne (A), Blekinge Archipelago (B), Lake Vänern (C) and Södermanland (D) where increased mortality had been observed (Table 1, Fig 1) [4,7,18]. The remaining 29 Herring gulls were caught between 13 June and 11 August the same year at three breeding sites in two regions of Iceland; Southwestern (E) and Eastern Iceland (F) (Table 1, Fig 1). They served as control birds as there were no signs of increased mortality at these sites. Detailed information on sampling locations (regions and breeding sites) and geographic coordinates are given in Table 1. All the birds in this study had been included in an earlier study on thiamine levels [7], and ten of the adult Herring gulls from Iceland had been used as healthy controls in a previous study on botulism [4].

**Table 1:** Summarized bird characteristics of the 75 Herring gulls *(Larus argentatus*) included in the study. For collection regions also refer Fig 1.

Collection regions^a^		A	B	C	D	E	F	Total
**No. of birds**		14	5	9	19	20	8	75
**Age**	*Juveniles*	0	0	9	10	10	0	29
	*Subadults*	1	2	0	0	0	0	3
	*Adults*	13	3	0	9	10	8	43
**Sex**	*Male*	3	3	4	7	7	7	31
	*Female*	11	2	5	12	13	1	44
**Condition**	*Medium*	9	1	6	16	20	8	60
	*Poor*	2	4	3	3	0	0	12
	*Emaciated*	3	0	0	0	0	0	3
**Clinical status**	*Healthy*	11	0	9	14	20	8	62
	*Diseased*	3	5	0	4	0	0	13

^a^Coordinates given according to the World Geodetic System 84 (WGS 84) (x_WGS84 y_WGS84):

A = Skåne, Spillepengen (13.05242 55.63046) and Pildammarna (12.99398 55.58935)

B = Blekinge Archipelago: Glipeskärvet (14.70540 56.11222), Vållholmen (14.53713 56.03464)

C = Lake Vänern: Galgen (13.88293 59.27972), Mellskär (13.86909 59.32820), Nypholmarna (13.88805 59.29809)

D = Södermanland: Måsklubbarna (17.39456 58.79456), Norra Gålklubben (17.57504 58.80335), Norra Kallskär (17.25201 59.30465), Sävö (17.47177 58.77240), Stenskären (17.35196 58.70909), Tärnskär (17.46029 59.32043), Västra Grässkär (17.21845 58.62167)

E = South Western Iceland: Midnesheidi (-21.95000 64.06667), Reykjanes (-22.69441 63.80861)

F = Eastern Iceland (Eastfjords): Djupivogus (-14.42444 64.68621).

Capture, handling and euthanasia by cervical dislocation were approved by the Swedish Ethical Committee for Scientific Experiments (protocol C57/4). Healthy and clinically abnormal birds were selected randomly and captured manually or by netting. Birds collected from Swedish locations were transported by car, and birds from Iceland were transported by car and air. An import permit was granted by the Swedish Board of Agriculture (Dnr 30-4205/04) and the transport complied with the International Air Transport Association (IATA) Live Animal Regulations. Feed and water were provided during transport. The clinical condition of all bird was assessed immediately before necropsy by a trained wildlife veterinarian. Clinical scores of '0' to '5' were used where 0 signifies a healthy bird and score '5' was given to moribund birds. The birds were divided into three age groups based on size and plumage colour and development [19]; juveniles (hatch year birds), subadults (sexually immature birds of at least one year of age), and adults (three years or older sexually mature birds). As described [7] some of the birds received thiamine (*n* = 13,50 mg/kg bw) or 0.9% saline solution (*n* = 13) intramuscularly (S2 Table) during confinement.

### Gross and microscopic pathology

The birds were weighed and necropsies were performed at Stockholm University by trained wildlife pathologists using a standard in-house protocol for wild birds of the National Veterinary Institute (SVA). Tissues for histopathology were fixed in 10% neutral buffered formalin, paraffin-embedded, sectioned at 4 μm in thickness, processed routinely and stained with haematoxylin and eosin (H&E). Tissues examined microscopically were: brain (proximal, mid and distal cerebral hemispheres, mid-cerebellum and medulla oblongata), spinal cord (transverse sections of lower neck, thorax and lumbosacral regions immediately proximal to the gelatinous body), thyroid gland, parathyroid gland, adrenal gland, trachea, lung, kidney, liver, pancreas, oesophagus, proventriculus, isthmus junction, gizzard, duodenum, mid-jejunum, ileum, caecum, colon, cloaca, bone marrow, spleen, gonad, myocardium (left atrium and ventricle), skeletal muscle (pectoral and thigh) and peripheral nerves (brachial plexus and ischiatic nerve). Thymus and bursa of Fabricius were sampled when present. Sections of spleen and gastrointestines from all birds were stained with alkaline Congo red and examined under a polarization microscope for amyloid. Additional tissues e.g. liver, kidney, myocardium, thyroid and brain were added in many birds with eosinophilic deposits. The degree of amyloidosis was estimated using a semi-quantitative scale from 1 (small but distinct focal deposits) to 4 (very pronounced systemic deposits). Tissues with granulomatous inflammation were stained with Ziehl-Neelsen, Periodic Acid Shiff (PAS) and Grocott stain, and peripheral nerves were stained with Luxol to reveal degenerative lesions. Perl stain was used to visualize iron deposits.

### Parasitology and cultures

Parasitological observations were made at necropsy and microscopically. Two samples of alcohol preserved parasite specimens were submitted for species identification to the Parasitic Worms Division, Department of Zoology, Natural History Museum, London, United Kingdom. Samples for bacteriology and mycology were cultured as described previously [4] when macroscopic lesions suggested infection.

### Characterization of protein AA from Herring gull amyloid

Amyloid fibrils were extracted from the liver of an adult Herring gull with severe amyloidosis from this study (bird no. 21, S2 Table). Extraction was made by repeated homogenization in 0.15 M NaCl, followed by distilled water as described [20]. The protein was purified by sequential gel filtration after solubilisation in 6 M guanidine HCl [21]. The amino acid sequence was determined by Edman degradation of the whole protein and of tryptic peptides as previously described [22].

### Statistical analysis

For differences between prevalence of amyloidosis in different areas Fischer’s exact test was used. For comparison between several groups, analysis of variance (ANOVA) with Tukey posttest was utilized. All calculations were performed with the Instat software. A p-value <0.05 was regarded statistically significant.

## Results

### Birds and clinical assessment

Table 1 summarizes data on age, sex, body condition, and clinical state of the birds from different areas. Individual gross findings, microscopic lesions, parasite findings and amyloid scores are presented in S1 and S2 Tables. Forty-eight out of the 75 birds were necropsied within 48 h post capture, and 26 birds were examined after three to five days except one bird that was kept for 17 days at a rehabilitation centre (bird no. 28, S2 Table) before necropsy. The mean time in captivity for adult birds from Sweden and Iceland were 2.7 ± 1.4 days and 2.6 ± 0.9 days, respectively (the Swedish gull kept in captivity for 17 days was excluded).

Eleven subadult or adult birds and one juvenile bird were classified as clinically abnormal at the time of necropsy (S1 and S2 Tables). A score of '0.5' or '1' was given to four birds showing inability to fly, weakness, listlessness and hypothermia (nos. 5, 11, 13 and 20, S2 Table). One subadult and one adult bird were assigned clinical scores of '2' (nos. 3 and 9, S2 Table). One juvenile, one subadult and four adult Herring gulls were assigned scores of '3' or '3.5' (no. 19, S1 Table, and nos. 1, 2, 4, 21 and 22, S2 Table). Two out of 12 affected birds were in normal body condition whereas the remaining were in poor condition or emaciated. Both sexes were represented among affected birds.

### Parasitology, pathology and culture results

**Fig 2:**
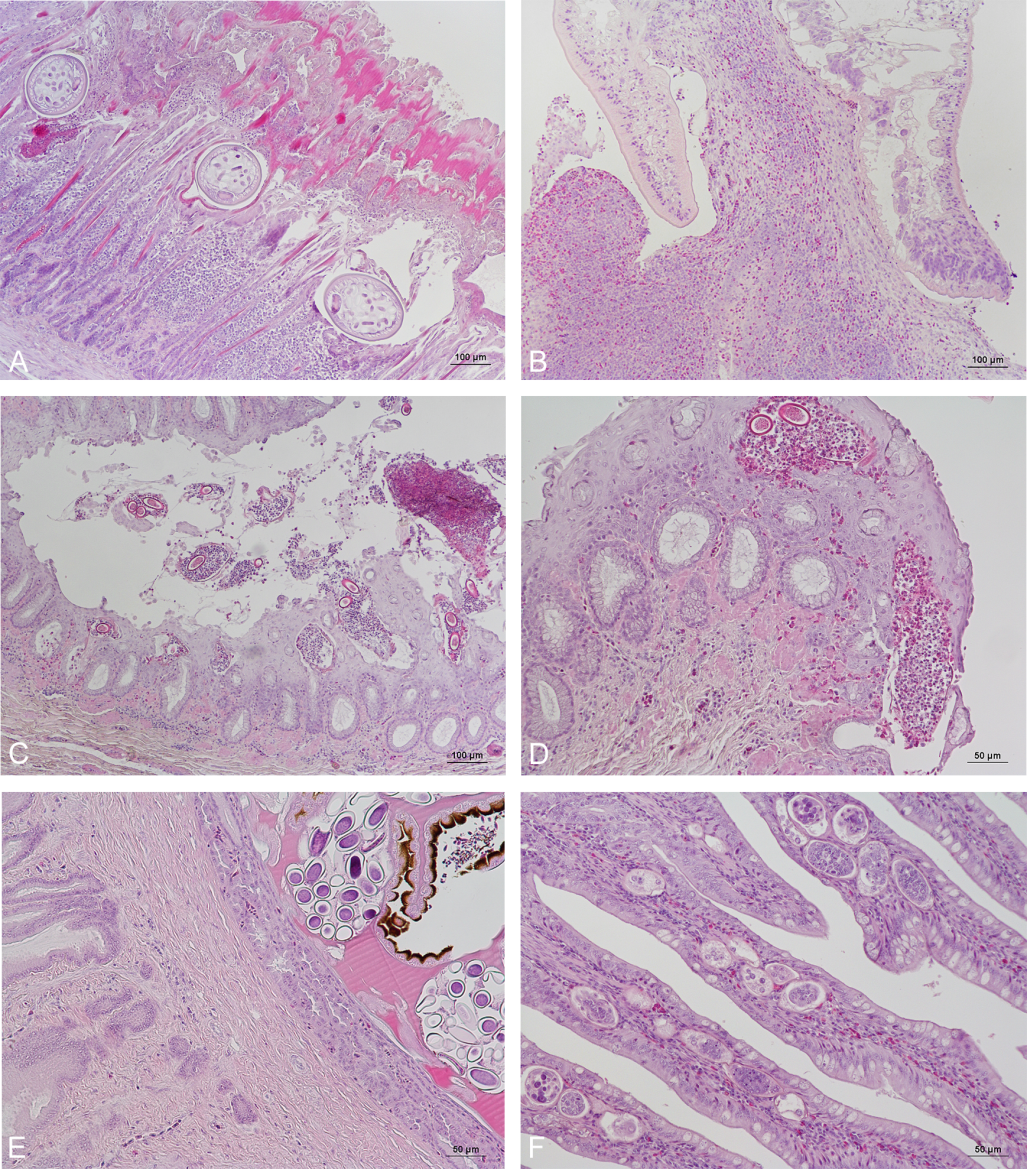
Microscopic parasite findings in Herring gulls (*Larus argentatus*). (A) Nematode infection in the gizzard with disruption of the koilin layer, focal necroses and lymphoplasmacytic inflammation (bird no. 12, S1 Table). H&E, bar 100 μm. (B) Trematodiasis (*Ichtyocotylarus platycephalus* (Creplin, 1825) of the bursa of Fabricius. Lymphocyte and heterophilic granulocyte infiltration and effacement of lymphoid follicles can be observed adjacent to rostral parasite structures (bird no. 6, S1 Table). H&E, bar 100 μm. (C–D) Oesophageal mucosa infected by *Capillaria* sp. Parasite eggs are present in the mucosa and in the lumen in association with inflammatory cells and necrotic debris (bird no. 11, S2 Table). H&E, bar 100 μm (C), 50 μm (D). (E) Proventricular mucosa with a trematode present in a submucosal gland. Note compression of glandular epithelium and absence of an inflammatory reaction (bird no. 6, S2 Table). H&E, bar 50 μm. (F) Jejunal villi with large numbers of parasite larvae (bird no. 17, S2 Table). H&E, bar 50 μm.

Individual gross pathology, microscopic diagnoses and parasite findings are shown in S1 and S2 Tables. Most juvenile birds including the one with clinical signs of disease (no 19, S1 Table) showed no gross pathology or microscopic lesions. Moderate to severe splenomegaly was observed grossly in two birds with reticuloendothelial cell hyperplasia. One of these also showed iron storage in the liver and erythrocytolysis and syncytia formation in the spleen of unknown aetiology. Microscopically, small peri-intestinal granulomas and a subcutaneous granuloma in the vent region of unknown cause were observed in one bird, respectively, focal pneumonia in two and mild non-suppurative perivascular encephalitis in two birds. Parasites of various classes and tissues were observed in many of the juvenile birds with no or mild signs of tissue lesions. This included coccidia in renal tissues and the intestines with a mild mixed inflammatory infiltrate in some cases, and cryptosporidia. Erosions and inflammation in the proventriculus and/or gizzard was present in three juvenile birds with microscopic evidence of intralesional nematodes in two cases (Fig 2A). Notably, six out of nine juvenile birds from Lake Vänern (collection area C) were diagnosed with bursal trematodiasis associated with the parasite *Ichtyocotylarus platycephalus* (Creplin, 1825) (Fig 2B). The parasites were attached to the bursal mucosa and obliterated the lumen. Microscopically, there was hyperplasia, multifocal squamous metaplasia and vacuolization of the bursal luminal epithelium. Interstitial inflammation with lymphocytes and heterophilic granulocyte infiltration and marked effacement of lymphoid follicles were observed at and around parasite attachment sites in the bursa. Extramedullary haematopoiesis was commonly observed in the liver, spleen, bursa of Fabricius and kidney and in some cases in the choroid plexus.

Some of the subadult or adult birds showed gross or microscopic findings that explained their poor condition and clinical disease. Among these was one bird with widespread mycobacteriosis in air sacs, lungs and thoracic wall (no. 11, S2 Table), and another bird with a chronic wound on the lower beak that may have prevented it from feeding (no. 13, S2 Table). Another bird showed multiple pathologic changes including granulomatous cholangiohepatitis adjacent to intrahepatic parasites as well as encephalitis (bird no. 9, S2 Table). There were also major findings in some birds that were scored as healthy such as in no. 6 (S2 Table), which had widespread pyogranulomatous pneumonia and air sacculitis, and air sac parasites, identified as the larid pentastome *Reighardia sternae* (Diesing, 1864) (S2 Table). Microscopically, large numbers of presumably secondary or opportunistic intralesional bacteria were observed, but bacterial and mycologic cultures revealed no growth.

Gastrointestinal helminths were observed grossly and/or microscopically in most subadult and adult gulls. *Capillaria* sp. was identified based on egg morphology in the oesophagus of five adult gulls from both Sweden and Iceland (Fig 2C and 2D), and six additional birds showed microscopic evidence of nematodes but without identifiable parasite eggs in the oesophagus (S2 Table). Additionally, there were 11 birds with fibrinosuppurative inflammation and/or lymphoplasmacytic perivasculitis in the oesophagus similar to the lesions present in parasite-infested gulls. Inflammation of the proventriculus and/or gizzard was another common diagnosis. Among the 17 affected adult or subadult birds, parasites were found microscopically in 12 (S2 Table). In some of these cases there was severe disruption of the koilin layer, haemorrhage and significant granulocyte and mononuclear infiltration in the epithelium and lamina propria. Other microscopic findings were trematodes within submucosal proventricular glands (Fig 2E), large numbers of embedded mucosal parasite larvae in the jejunum (Fig 2F) and sarcocystosis of skeletal musculature and myocardium (*n* = 11). Acute to subacute rhabdomyolysis of leg and pectoral musculature and occasionally necrosis of myocardial fibres was present in several juvenile and adult birds, presumably associated with captivity (capture myopathy) (S1 and S2 Tables). No significant bacterial or fungal species were isolated from granulomas in bird nos. 6 and 11 (S2 Table), and α-streptococci were isolated from the liver in bird no. 9 (S2 Table) in which miliary pinpoint-sized yellow foci had been observed at necropsy.

### Occurrence and degree of amyloidosis

Amyloid was detected in 26 out of 28 adult Herring gulls (93%) and in 3 out of 3 (100%) subadults sampled in Sweden. No amyloid was seen in tissues from juveniles. Summarised semi-quantitative amyloid scores in subadult and adult Herring gulls are shown in Table 2, and individual amyloid scores are shown in S1 and S2 Tables. All the 14 animals from the Malmö area (of which 13 were from breeding site Spillepengen) were affected as were 10 of the 12 (83%) Herring gulls from other locations in Sweden. In contrast, only 6 birds out of 18 adult birds from Iceland were affected (33%). The difference between Swedish and Icelandic animals was statistically significant (*p*<0.001).

**Table 2:** Summary of semi-quantitative amyloid scores in subadult and adult Herring gulls (*Larus argentatus*). For more information on collection regions refer to text, Fig 1 and Table 1.

Collection region	No of birds with amyloid /total number	Amyloid score	
		Range	Mean
**A**	14/14	1–4	2.3
**B**	5/5	1	1.0
**D**	5/7^a^	0–4	1.6
**E**	2/9	1–3	0.4
**F**	3/8	1	0.4

^a^Spleen missing in one negative bird; One 2+ bird with exceptional time of capture (17 days).

The severity of amyloidosis differed significantly between Swedish and Icelandic birds; 1.9± 1.2 and 0.4±0.8, p<0.001), respectively. This difference depended on the pronounced degree of amyloidosis in birds from the Malmö area (2.4±1.1), which differed significantly from birds from both Icelandic areas (*p*<0.01 for both comparisons), while there was no significant difference in degree of amyloidosis between birds from the remaining regions of Sweden and from Iceland. Since captivity has been reported to influence development of AA amyloidosis in gulls, the time from capture to euthanasia was compared between Swedish and Icelandic birds. This time was 2.7±1.4 and 2.6±0.9 days, respectively (p>0.05).

### Distribution of amyloid

**Fig 3:**
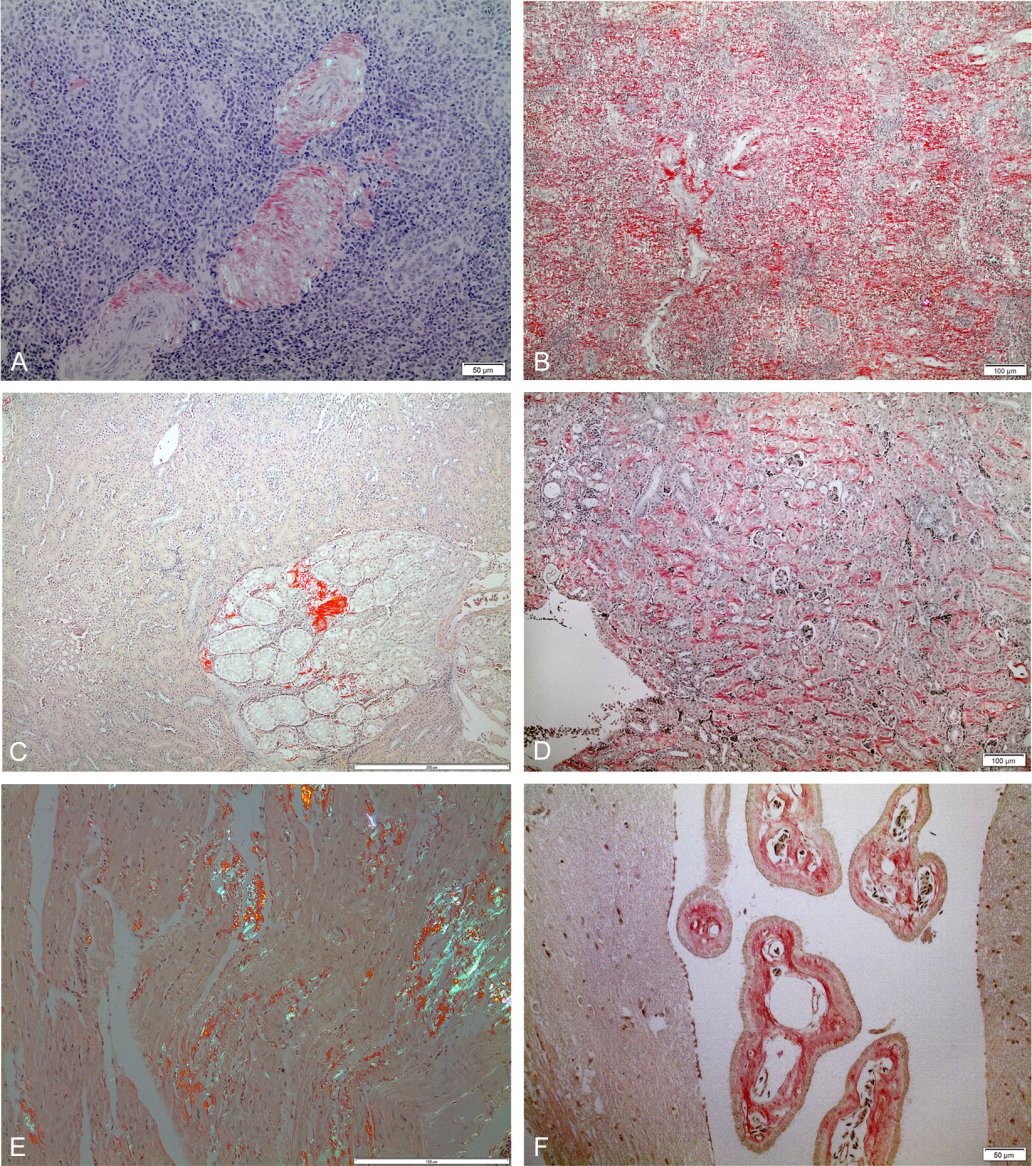
Microscopic amyloid findings in Herring gulls (*Larus argentatus*). (A) Amyloid deposits mainly in arterial adventitia of the spleen (bird no. 27, S2 Table, amyloid score 1). Congo red, crossed polars, bar 50 μm. (B) Massive diffuse amyloid infiltration throughout the splenic parenchyma (bird no. 6, S2 Table, amyloid score 4). Congo red, bar 100 μm. (C) Small, localized deposits around some kidney tubules and in walls of small arteries. (bird no. 33, S2 Table, amyloid score 3). Congo red, bar 200 μm. (D) Diffusely spread amyloid deposits interstitially in the kidney. Note glomeruli virtually free of amyloid (bird no. 9, S2 Table, amyloid score 4). Congo red, bar 100 μm. (E) Pronounced but patchy and mainly interstitial deposits of amyloid in the myocardium (bird no. 11, S2 Table, amyloid score 4). Congo red, crossed polars, bar 100 μm. (F) Brain was free of amyloid in all birds except for the choroid plexus, which is outside the blood-brain barrier. Amyloid is present in vessel walls (bird no. 9, S2 Table, amyloid score 4). Congo red, bar 50 μm.

Amyloid in the investigated Herring gulls had the usual affinity for Congo red and exhibited bright birefringence when examined in a polarization microscope. Even very small deposits were easily recognized. Like in other vertebrates, deposits were widely distributed, and in birds with pronounced disease most organs were involved except for the brain and the spinal cord. The most pronounced amyloid infiltration was usually observed in the spleen. The splenic distribution varied from vascular deposits (Fig 3A) of varying degree to a diffuse, evenly distributed involvement of the parenchyma (Fig 3B). When very little amyloid appeared in the spleen this was confined to vessel walls and to the capsule. The liver in affected birds exhibited vascular deposits. Only in a few birds with very pronounced amyloidosis, deposits were more diffusely distributed in the liver parenchyma. In most birds with amyloidosis, deposits were found in the kidneys, most commonly confined to vessel walls (Fig 3C). In kidneys with pronounced amyloidosis, peritubular amyloid infiltration was conspicuous while glomerular involvement was minimal (Fig 3D). In general, only small amounts of amyloid were seen in the myocardium. In some animals with severe amyloidosis however, relatively large deposits were seen scattered within the myocardium (Fig 3E). In animals with pronounced amyloidosis, large amounts of amyloid were found in pancreatic vessel walls, sometimes with interstitial deposits in the exocrine parenchyma, but islets of Langerhans were occasionally also infiltrated. The thyroid gland contained only little amyloid. Lung and brain were free of amyloid except for heavy involvement of choroid plexus in a few birds (Fig 3F).

**Fig 4:**
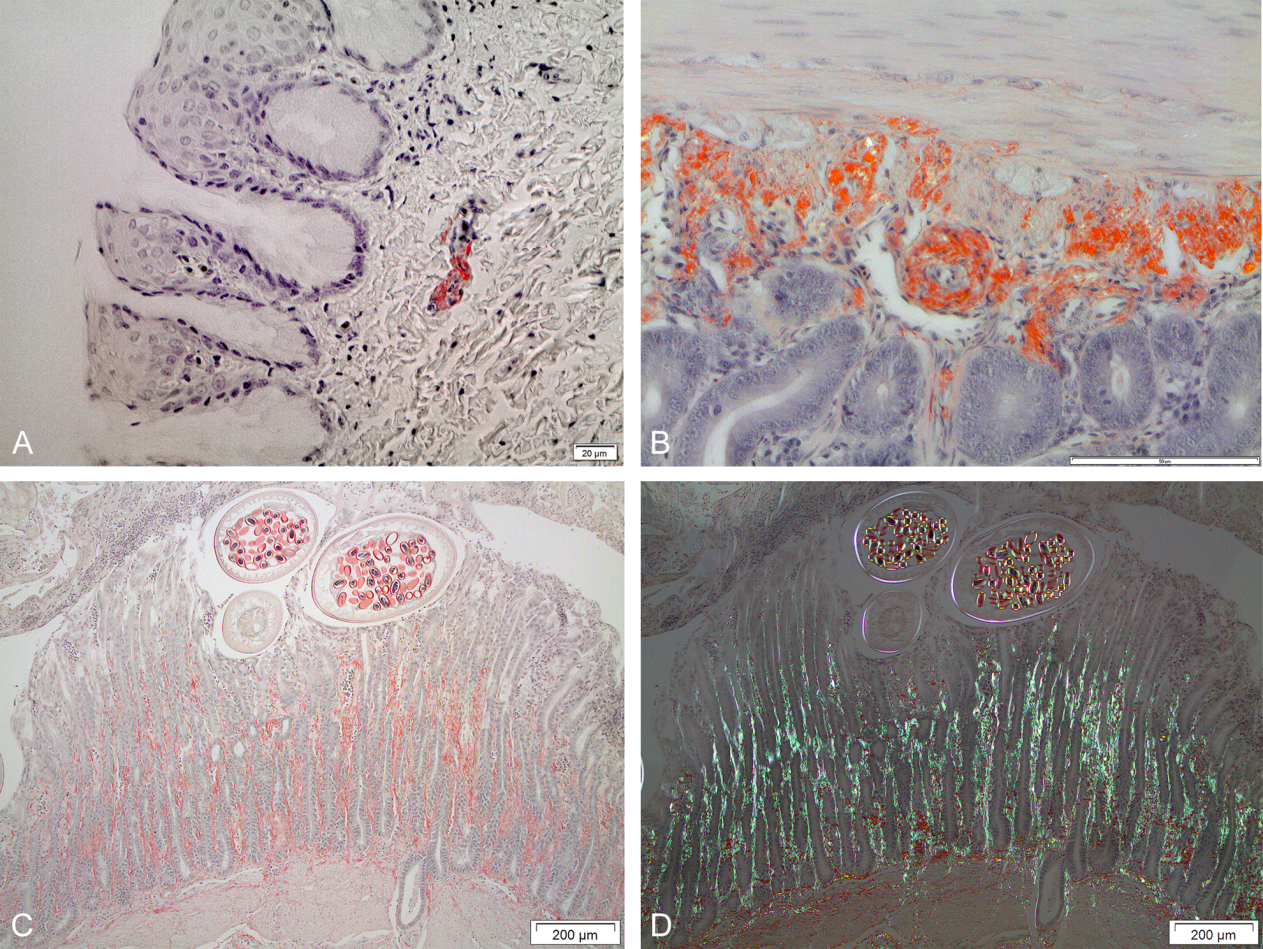
Microscopic amyloid findings in Herring gulls (*Larus argentatus*). (A) Amyloid in a small artery in the oesophageal submucosa (bird no. 29, S2 Table, amyloid score 1). Congo red, bar 20 μm. (B) Extensive amyloid deposits in small intestinal vessel and lamina propria in basal position in the mucosa. Some amyloid is also seen in between mucosal glands (bird no. 21, S2 Table, amyloid score 4). Congo red, bar 50 μm. (C–D) Extensive amyloid deposits are seen in small vessel walls almost throughout the full thickness of the gizzard mucosa. Note three parasites in the outer part of the mucosa. Note also congophilia in the parasite eggs (bird #11, S2 Table, amyloid score 4). Congo red, in C) ordinary light and in D) crossed polars, bars 200 μm.

Notably, the gastrointestinal (GI) tract was involved in virtually every bird with amyloidosis. Deposits were found throughout the entire GI tract but varied in severity between birds. The smallest (and probably earliest) deposits were identified in the walls of thin arteries or arterioles in lamina propria, just inside the muscularis mucosae (Fig 4A). Amyloid was often also seen in thin vessels running perpendicularly against the lumen. Very often, muscularis mucosae was involved, even in cases with only mild amyloidosis (Fig 4B). When deposits were more severe, a widely spread vascular involvement was seen in all layers of the proventriculus, gizzard and intestine. Very often, thin amyloid streaks occurred in the lamina propria mucosae, following vessels up to superficial parts of the mucosa (4C), sometimes in close proximity to intestinal parasites. Outer walls of parasite eggs had affinity for Congo red and showed a greenish birefringence (Fig 4D). Since amyloid often was found close to the GI mucosal surface, a search for amyloid material within the GI lumen was performed. Congophilic material, obviously originating from feed was often identified. However, in one gull with severe amyloidosis (bird no. 11, S2 Table), typical amyloid material was identified within the intestinal lumen.

### Characterization of Herring gull protein AA

**Fig 5:**
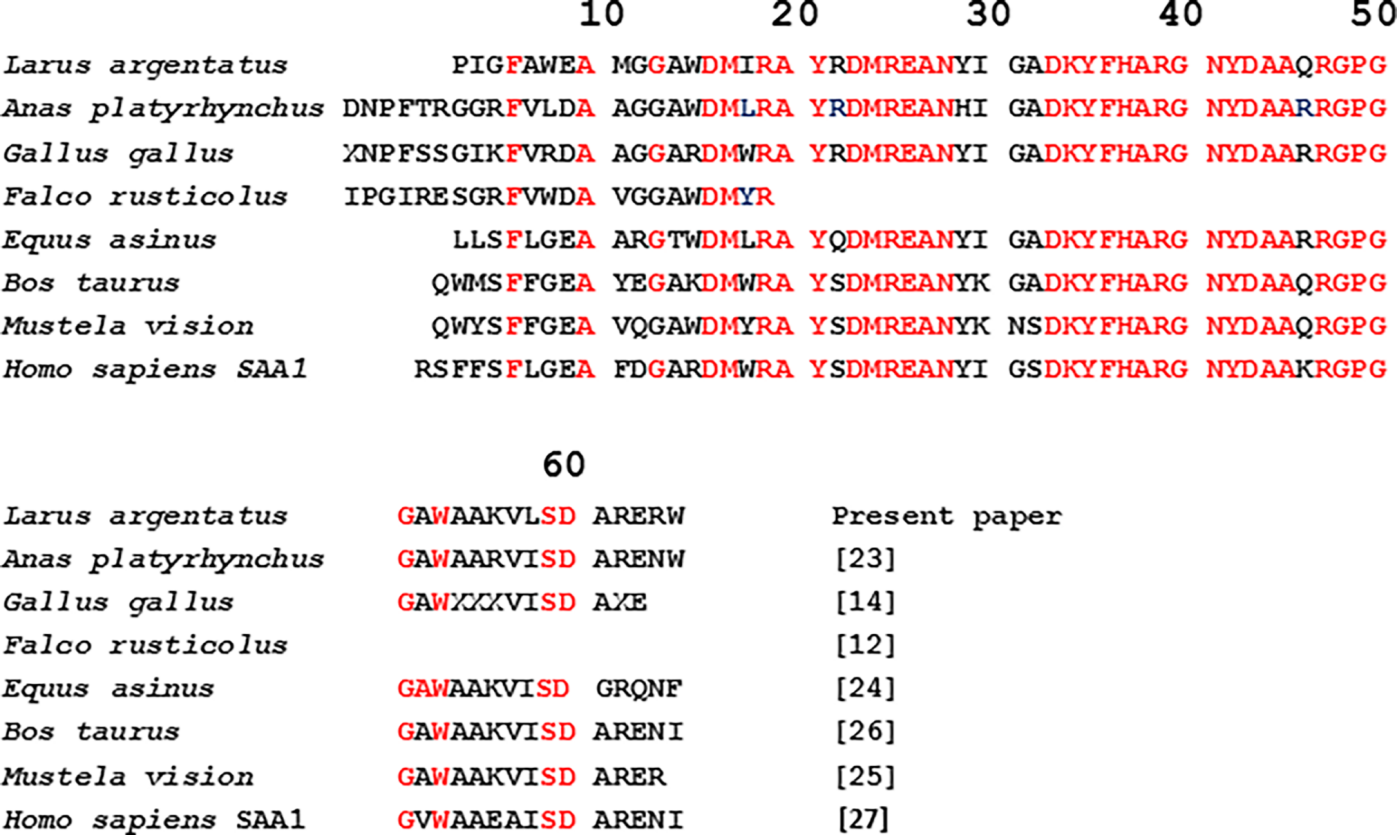
N-terminal amino acid sequence of Herring gull (*Larus argentatus*) protein AA compared with the same region of protein AA of some birds and mammals. Identical amino acid residues are shown in red.

Gel filtration of dissolved amyloid resulted in one large retarded peak, corresponding to protein AA. Edman degradation revealed a free N-terminus. The amino acid sequence of Herring gull protein AA from this study is shown in Fig 5 together with those from several avian and mammalian species [12,14,23,24,25,26,27]. As seen, the sequence was highly conserved but varied significantly at the N-terminus to the completely conserved F6 (numbering according to the human AA sequence). The Herring gull SAA/AA molecule differed at the N-terminus from the other avian species from which protein AA sequence has been determined and it lacked the six amino acid residue extension [12,14,23]. The Herring gull amino acid sequence also was unusual by the presence of proline 3, glycine 5, tryptophane 8 and methionine 11, but it had the completely conserved regions between residues 33–45 and 47–51 found in SAA/AA of all other studied avian and mammalian species. The amino acid sequence was established to residue number 60 (numbering according to the human protein sequence) where a major protein AA species ended. Somewhat longer AA species were probably present but no more sequence data were obtained.

## Discussion

### Pathology and parasitology

Among clinically affected subadult and adult birds in this study, several were diagnosed with severe chronic inflammatory processes (S2 Table) that could putatively explain their clinical signs. However, no clear causes of disease were identified in other affected birds. Tests for the detection of botulinum toxin were not included in this study, therefore it remains a possibility that botulism was the cause of disease in at least some birds. Also, thiamine deficiency was previously reported in some of the birds in this study [7]. Various disease processes were also identified in some birds scored as healthy. One of the most common findings was amyloidosis.

A wide variety of parasites were observed grossly and/or microscopically in the investigated birds of this study. Several earlier studies have reported complex parasite communities in different gull species from various habitats [4,28,29]. There is, however, limited knowledge on detrimental effects of parasites on the fitness and reproductive success in free-living wild seabirds. Even less is known about variations of parasite load and species distribution among different Herring gull populations. However, a previous study suggested that Glaucous gulls (*Larus hyperboreus*) that received anti-parasitic treatment had lower blood levels of organochlorines and improved nesting success compared to untreated birds [30] and in another study, there were negative effects on body condition from high parasite loads [31]. In our study, several parasite findings such as oesophageal capillariosis, nematode infection in the proventriculus and/or gizzard, the finding of the pentastomid *Reighardia sternae* in association with a severe granulomatous air sacculitis (bird 6, S2 Table) as well as bursal trematodiasis with bursal inflammation and follicular atrophy in nestlings from one geographical area, suggested that at least some parasites may have significant detrimental effects in Herring gulls. However, it seems unlikely that parasite infections would explain the mortality noted in the Baltic Sea population of Herring gulls.

### Amyloidosis

We investigated the occurrence and distribution of amyloidosis in this study. Amyloidosis of varying degrees of severity occurred in most adult birds captured in Sweden (93%), but at lower occurrence in birds from Iceland (33%). As expected juvenile birds were not affected, but amyloid was present already in 1–2-year-old subadult birds. We purified the major amyloid fibril protein and characterized the major fibril protein AA. Amino acid sequence analysis showed that the major part of protein AA was a 63 residues long N-terminal fragment of SAA. Cleavage of SAA has been postulated to be of importance in amyloidogenesis in mammals [32]. On the other hand, protein AA in the duck has been determined to consist of full-length SAA molecules [23]. Consequently, importance of protein cleavage in amyloid A pathogenesis is still unclear. It is also notable that the amino acid sequence is remarkably conserved between birds and mammals.

Amyloidosis in Herring gulls has been previously reported from both confined and free-living wild birds [4,8]. One of the salient findings of this study was that the occurrence (93%) of amyloidosis among adult Swedish Herring gulls was remarkably high, and comparable only to figures from a zoological collection of captive birds [33]. On the other hand, the studied birds from Sweden were not randomly obtained because they originated from sites where increased mortality had occurred and some of the birds displayed signs of disease. Gulls caught from one area (A) appeared to be more severely affected by amyloidosis compared to other gulls in this study. All but one bird were caught from the same location (Spillepengen), an area consisting of reclaimed recreational land, a water treatment plant, a former refuse dump and waste recycling facilities. These findings warrant further investigation as there is currently no explanation as to why gulls in Sweden seemed more prone to develop amyloidosis compared to gulls in Iceland.

Like in other animals, most organs were affected in the Herring gulls with pronounced amyloidosis in this study. The material was not ideal for the study of development and spread of deposits but some conclusions could be drawn. The spleen was always involved in birds with tiny amounts of amyloid infiltration. In contrast to mice, in which the first amyloid appears in the perifollicular zone, the smallest deposits in Herring gulls were found in walls of small splenic arteries as well as a very thin rim in the capsule. In birds with such small and probably early deposits, thin blood vessels in the intestinal lamina propria were usually also affected. Therefore, the first amyloid in gulls seems to appear in association with smooth muscle cells while in mice, marginal zone macrophages play a crucial role [34,35]. Similar results were reported by Hoffman and Leighton [8] who found that control gulls shot in the field had small amyloid deposits in splenic arteries. The renal distribution in birds seems to differ from that of mammals with most severe amyloid infiltration in interstitial location and little in glomeruli.

In mammals, AA amyloidosis appears after prolonged high expression of SAA, usually as a consequence of chronic inflammatory diseases [36]. The situation in birds is probably the same, although it has not been much studied. In the present study, a majority of the birds with the highest amyloid score (such as bird nos. 6, 9 and 11, S2 Table) were diagnosed with chronic widespread inflammatory processes, which is in support of earlier research. However, no obvious cause for amyloid deposition was found in others. The significance of parasite-associated inflammation as a trigger for amyloidosis is unknown in birds but should be further investigated. In particular, nematode infection and chronic inflammation were commonly observed in the oesophagus, proventriculus and gizzard among the adult birds, but less often in specimens from younger gulls. Previously, nematodes have been shown to induce amyloidosis in hamsters [37]. Chronic infections and parasitic infestation were found in one study of avian amyloidosis in which, however, a large proportion of affected birds lacked such diseases [38]. Another potentially important contributing factor could be the high level of environmental pollution present in the Baltic Sea, which could possibly trigger the onset of an acute phase response and amyloid deposition in birds. Although there is no proof today of such an association, the higher proportion of severely affected birds in the Southern Baltic Sea in this study compared to inland sampling location and in Iceland where the level of pollution can be assumed to be lower warrants further investigation. Stress also seems to be a cause of acute phase response in captive birds [8]. Hoffman and Leighton [8] reported that wild Herring gulls developed systemic amyloidosis upon capture within eight days. Almost all gulls in this study were collected alive and kept in captivity during transport to the laboratory for a period of 1–5 days. An important consideration is thus whether this is one reason for the high prevalence of the disease in Swedish birds. However, Icelandic birds were kept alive for a period almost identical to that of Swedish gulls and still had a lower incidence of amyloidosis. Thus, although some effect of stress associated with captivity may have contributed, it is unlikely that it is the primary explanation for amyloidosis. This together with other potential causes of stress present pre-capture to varying degrees at different locations, such as large bird colony size with overcrowding, microbial contamination, parasitism and competition with other species at breeding sites, limited feed resources and environmental pollution, should be considered in future studies.

AA amyloidosis has properties reminiscent of prion diseases (for review, see [39]). Susceptible mouse strains as well as other mammals usually require several weeks before any amyloid can be found despite persistent very high plasma SAA concentration [40]. On the other hand, mice which have received a seed (sometimes referred to as ‘amyloid enhancing factor’ (AEF) in association with an inflammatory stimulus, develop amyloidosis much more rapidly. The seed is an extract containing amyloid fibrils, which is believed to act by a prion-like mechanism and is active both when given intravenously and by the oral route [41,42]. Interestingly, a seed can be given as a single dose to an animal up to at least six months before inflammation is induced and still be efficient [41]. This suggests that fibrillary seeding material can be stored in tissues for a very long time without degradation. Furthermore, in mice, AA amyloidosis may resolve if inflammation ceases. Such animals have been shown to be in a ‘primed state’ and very rapidly redevelop the disease if inflammation is reinduced [40,43]. AA amyloidosis may be transmitted orally to susceptible mammals and chickens [39,44] including by the faecal-oral route in the cheetah [45]. The pronounced involvement of the intestinal mucosa with amyloid deposits sometimes seen quite close to or even within the intestinal lumen in the Herring gulls in this study is therefore of particular interest. Our findings support extrusion of amyloid into the intestinal lumen as a route of amyloid excretion. The Herring gull is a long-lived, colony-breeding coastal bird species with omnivorous, scavenging and opportunistic feeding habits. Their diet includes fish, crustaceans, bivalves, earthworms, insects, dead and diseased birds, eggs and nestlings and refuse. Given the omnivorous feeding habits of Herring gulls and the tendency to live in large and densely populated colonies with nests on the ground it seems likely that these birds now and then consume amyloid-contaminated feed. Another possible mechanism of seed transmission, although highly speculative, is that fibrils are transmitted already at the nestling stage since juvenile gulls are fed by regurgitating parents. Consumption of regurgitated feed is also a component of courtship among breeding pairs. A primed state may therefore commonly exist in this particular species. Such a scenario would, together with inflammatory processes, captivity, and/or other chronic stress explain the rapid onset of amyloid deposition and the high prevalence of AA amyloidosis in Herring gulls from Sweden.

## Conclusions

Small amyloid deposits probably are of no clinical significance in Herring gulls and other birds. However, animals with such pronounced deposits as were observed in at least seven of the birds from the Malmö area in this study, most likely were severely affected by the disease. Given the severe amyloid infiltration in the gastrointestinal tract of such animals, it is not unlikely that they may have suffered from malabsorption. Amyloidosis may therefore have contributed to the thiamine deficiency previously reported from Swedish Herring gulls [7]. Our results thus suggest that amyloidosis could be a contributing factor to declining Herring gull populations in the Baltic Sea habitat. For reasons that have yet to be determined, gulls from Sweden were more commonly affected that those from Iceland. We suggest that future studies should focus on potential factors involved in the development of amyloidosis such as stress, parasitism and inflammatory stimulation, immunomodulators and prion-like seeding.

## Supporting information

S1 Table Main findings in juvenile Herring gulls (*Larus argentatus*) from Sweden and Iceland. (XLSX)

S2 Table List of main findings in subadult and adult Herring gulls (*Larus argentatus*) from Sweden and Iceland. (XLSX)
